# Acute Pancreatitis after Kidney Transplantation

**DOI:** 10.1155/2012/768193

**Published:** 2012-05-07

**Authors:** Mithat Tabakovic, Nermin N. Salkic, Jasmina Bosnjic, Ervin Alibegovic

**Affiliations:** ^1^Department of Nephrology, Dyalisis and Transplantation, Internal Medicine Hospital, University Clinical Center Tuzla, Trnovac bb, 75000 Tuzla, Bosnia And Herzegovina; ^2^Department of Gastroenterology and Hepatology, Internal Medicine Hospital, University Clinical Center Tuzla, Trnovac bb, 75000 Tuzla, Bosnia And Herzegovina; ^3^Pulmonary Diseases Hospital, University Clinical Center Tuzla, Trnovac bb, 75000 Tuzla, Bosnia And Herzegovina

## Abstract

Acute pancreatitis is a rare but life-threatening complication in patients with transplanted kidney. The incidence of acute pancreatitis after kidney transplantation ranges from 2% to 7%, with mortality rate between 50 and 100%. We report a case of a female patient aged 46 years, developing an interstitial acute pancreatitis 8 years following a renal transplantation. The specific aethiological factor was not clearly established, although possibility of biliary pancreatitis with spontaneous stone elimination and/or medication-induced pancreatitis remains the strongest. Every patient after renal transplantation with an acute onset of abdominal pain should be promptly evaluated for presence of pancreatitis with a careful application of the most appropriate diagnostic procedure for each individual patient.

## 1. Introduction

Acute pancreatitis after renal replacement was first described by Starzl in 1964 [1]. It is luckily a rare but dangerous and potentially life-threatening complication of kidney transplantation. Etiological causes are very hard to determine precisely and usually combination of various factors is incriminated. We describe a case of acute pancreatitis in a female patient 8 years after renal transplantation.

## 2. Case Report

A female patient, 46 years old, was admitted to our hospital due to gastrointestinal symptoms that started 3 days prior to hospitalisation. She complained of vomiting, nausea, a strong epigastric pain radiating to both flanks, loss of appetite, and general malaise. She did not have fever nor any changes in stool frequency and consistency.

She was diagnosed with end stage renal disease 11 years ago with a chronic (membranous) glomerulonephritis as a cause. For three years she was treated with dialysis until finally, 8 years ago, she had a living donor transplantation of kidney donated by her mother. A year following the transplantation she had another surgical intervention—a laparoscopic cholecystectomy without any particular perioperative or postoperative problems. She did not have any positive history for alcohol consumption.

Immunosuppressive protocol at the time of admission included combination of cyclosporine, mycophenolate mofetil and prednisone with regular followup by her nephrologist and without any signs of graft rejection or dysfunction. During her posttransplant period, there were no episodes that required administration of OKT3 antithymocyte globulin.

Her physical exam revealed moderate tenderness of her abdomen, particularly in her epigastric region.

Initial laboratory tests revealed a significant elevation of serum and urine amylase levels (2439 IU/L and 18733 IU/L, resp.), with an elevated levels of AST (408 IU/L), ALT (539 IU/L), ALP (307 IU/L), total (31.6), direct (11.9), and indirect (19.7) bilirubin. Her creatinine (171) and BUN (10.9) levels were also slightly elevated. Her leukocyte count and CRP level were normal, as were the other biochemical parameters. Serum calcium levels and parathyroid hormone and cyclosporine levels were also in normal range.

Serological status regarding cytomegalovirus, Epstein-Barr virus, herpes simplex virus, and varicella zoster virus infection was negative for acute infection. PCR tests for CMV infection and viral hepatitides were also negative.

**Figure 1:**
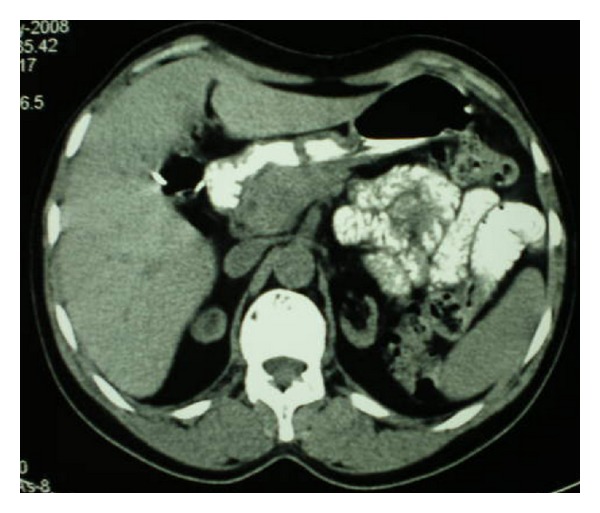
Computerised tomography of pancreas depicting enlarged and inhomogenous head of the pancreas.

This clinical and biochemical pattern was suggestive for acute pancreatitis so we proceeded with native CT scan due to a possible danger of contrast-induced nephropathy in the setting of already elevated creatinine. The CT scan revealed a slightly enlarged pancreas with inhomogeneous head and without peripancreatic fluid—a finding consistent with mild interstitial pancreatitis of the head of the pancreas (Figure 1).

There were no conclusive findings on common bile duct. Since the patient had cholecystectomy and lab parameters suggested a possible biliary aetiology, we performed an endoscopic ultrasound which confirmed pancreatitis but ruled out possible presence of choledocholithiasis. Transabdominal ultrasound was also conclusive with interstitial pancreatitis while renal graft had normal echosonographic appearance and Doppler findings.

We concluded that patient had interstitial pancreatitis and treated her with standard medical care, while feeding and treating her by way of parenteral route for 48 hours. We administered IV Pantoprazol in 80 mg per day divided in two doses, without antibiotic treatment, while continuing immunosuppresive regimen with exception of prednisone. After 48 hours we started with peroral treatment with quick improvement of patient condition and her discharge was after 10 days of hospitalisation.

## 3. Discussion

The incidence of acute pancreatitis after renal replacement treatment ranges from 2% to 7% with mortality rates ranging from 50% to 100% [2–6]. Theories about the aetiology of pancreatitis after renal transplantation are numerous. Among many, medications used in immunosuppressive treatment remain the strongest suspects [7]. Nevertheless, one cannot neglect the usual cause such as bile duct stones, alcohol, and hyperlipidaemia. Among the other possibilities, there is also a hyperparathyroidism, especially in patients with long-term dialysis prior to renal replacement treatment [8, 9]. There is also an important possibility of viral infection in these patients with cytomegalovirus as frequent suspect [10]. Nevertheless, very often, the aetiology of the acute pancreatitis is not always identifiable in these patients [11].

Due to a immunosuppressive treatment, acute pancreatitis in patients following renal transplantation has altered pathophysiology that leads to a higher mortality risk compared to a nontransplant population [7]. There are speculations that impaired acute phase response in posttransplant patients is the possible reason for this [12]. There are reports suggesting that classical symptoms and laboratory findings are often absent, which may cause diagnostic difficulty and may be the consequence of impaired acute response [11].

It is therefore imperative to retain the high level of suspicion for every patient with acute onset of abdominal pain, especially if they have one or more known risk factors. There are suggestions that CT scanning should be the test of choice in clinically suspected cases, even with the proposed gradation of severity based on CT appearance [7, 11]. The diagnostic yield of such approach is indeed great, however, one must not forget that we are dealing with a patients with transplanted kidney where the possible danger of contrast-induced nephropathy must be carefully weighted [13]. Therefore, other diagnostic modalities such as endoscopic ultrasound may be considered. Due to a nature of the procedure, this particular imaging modality provides an accurate assessment of pancreatic gland, its surrounding, and biliary system.

It is very hard to establish the aethiology of pancreatitis in our particular patient. There is of course a possibility of common bile duct calculi with spontaneous elimination—a scenario frequently seen in patients after cholecystectomy. Other possible option is the medication-induced pancreatitis perhaps with the cyclosporine as an incriminating agent. It is a fortunate fact that our patient had a mild form of pancreatitis without necrosis and subsequent complications which lead to the relatively quick recovery and normalisation of laboratory parameters.

In conclusion, every patient after renal transplantation with an acute onset of abdominal pain should be promptly evaluated for presence of pancreatitis with a careful application of the most appropriate diagnostic procedures for each individual patient. Unnecessary contrast-enhanced procedures in patient after renal replacement treatment may endanger the graft and thus further complicate a clinically grave situation caused by pancreatitis.

## References

[1] Starzl TE (1964). *Experience in Renal Transplantation*.

[2] Burnstein M, Salter D, Cardella C, Himal HS (1982). Necrotizing pancreatitis in renal transplant patients. *Canadian Journal of Surgery*.

[3] Corrodi P, Knoblauch M, Binswanger U (1975). Pancreatitis after renal transplantation. *Gut*.

[4] Fernandez-Cruz L, Targarona EM, Cugat E, Alcaraz A, Oppenheimer F (1989). Acute pancreatitis after renal transplantation. *British Journal of Surgery*.

[5] Kenmochi T, Asano T, Shimada H, Ochiai T, Isono K (1992). Clinical and experimental studies of acute pancreatitis after renal transplantation. *Transplantation Proceedings*.

[6] Penn I, Durst AL, Machado M (1972). Acute pancreatitis and hyperamylasemia in renal homograft recipients. *Archives of Surgery*.

[7] Slakey DP, Johnson CP, Cziperle DJ (1997). Management of severe pancreatitis in renal transplant recipients. *Annals of Surgery*.

[8] Sitges-Serra A, Gores P, Hesse U (1986). Serum calcium as an early indicator for surgical treatment of hyperparathyroidism after renal transplantation. *World Journal of Surgery*.

[9] Stephani J, Akinli AS, Von Figura G (2011). Acute pancreatitis in a patient with hypercalcemia due to tertiary hyperparathyroidism. *Zeitschrift fur Gastroenterologie*.

[10] Sinha S, Jha R, Lakhtakia S, Narayan G (2003). Acute pancreatitis following kidney transplantation—role of viral infections. *Clinical Transplantation*.

[11] Taylor K, Sinha S, Cowie A, Babbs C, Reeve R, Kalra PA (2009). Challenges in diagnosing acute pancreatitis in renal transplant patients. *Clinical Transplantation*.

[12] Edwards CM, Morgan JDT, Tilsed JVT, Donnelly PK (1992). Impaired acute phase response: a risk factor for life-threatening posttransplant pancreatitis?. *Transplantation Proceedings*.

[13] Elicker BM, Cypel YS, Weinreb JC (2006). IV contrast administration for CT: a survey of practices for the screening prevention of contrast nephropathy. *American Journal of Roentgenology*.

